# Identification of Bacterial Protein *O*-Oligosaccharyltransferases and Their Glycoprotein Substrates

**DOI:** 10.1371/journal.pone.0062768

**Published:** 2013-05-03

**Authors:** Benjamin L. Schulz, Freda E. C. Jen, Peter M. Power, Christopher E. Jones, Kate L. Fox, Shan C. Ku, Joanne T. Blanchfield, Michael P. Jennings

**Affiliations:** 1 Institute for Glycomics, Griffith University, Gold Coast, Queensland, Australia; 2 School of Chemistry and Molecular Biosciences, The University of Queensland, Brisbane, Queensland, Australia; 3 Department of Paediatrics, University of Oxford, Oxford, Oxfordshire, United Kingdom; 4 School of Science and Health, The University of Western Sydney, Penrith, New South Wales, Australia; University of Helsinki, Finland

## Abstract

*O*-glycosylation of proteins in *Neisseria meningitidis* is catalyzed by PglL, which belongs to a protein family including WaaL O-antigen ligases. We developed two hidden Markov models that identify 31 novel candidate PglL homologs in diverse bacterial species, and describe several conserved sequence and structural features. Most of these genes are adjacent to possible novel target proteins for glycosylation. We show that in the general glycosylation system of *N. meningitidis*, efficient glycosylation of additional protein substrates requires local structural similarity to the pilin acceptor site. For some *Neisserial* PglL substrates identified by sensitive analytical approaches, only a small fraction of the total protein pool is modified in the native organism, whereas others are completely glycosylated. Our results show that bacterial protein *O*-glycosylation is common, and that substrate selection in the general *Neisserial* system is dominated by recognition of structural homology.

## Introduction

Protein glycosylation occurs in all domains of life, where it is important in protein folding, stability and function. The presence of bacterial glycoproteins has long been known, but recent years have shown a dramatic increase in reports of protein glycosylation in diverse bacteria [1], [2]. Genes encoding these glycoproteins are typically encoded in operons that include a glycosyltransferase and a single acceptor glycoprotein. Several recent reports have described general glycosylation systems in Gram-negative bacteria, including *Neisseria meningitidis* [3], *Neisseria gonorrhoeae* [4], *Campylobacter jejuni* [5], [6] and *Bacteroides fragilis* [7]. In these general systems, a single glycosyltransferase or oligosaccharyltransferase enzyme modifies multiple different substrate proteins and the genes encoding the enzyme and substrate proteins are typically not closely linked on the genome.

The pathogenic *N. meningitidis* is the causative agent of meningococcal meningitis and septicaemia and is a worldwide health burden. *N. meningitidis* has a general system for protein *O*-glycosylation, which modifies PilE (Pilin), the major adhesin of *N. meningitidis* [8], and AniA, a surface exposed nitrite reductase [3]. A model has been developed for protein O-glycosylation in this system, which is similar to *wzy*-dependent O-antigen biosynthesis in Gram-negative bacteria [9]. In this model, a glycan to be transferred to protein is assembled on a diphosphate-polyprenyl lipid carrier on the cytoplasmic face of the inner membrane by the sequential action of glycosyltransferases PglB, PglA, PglE and the acetyltransferase PglI. *N. meningitidis* can have different repertoires of glycosyltransferases, with PglB2, PglG and PglH also present in some isolates [10], [11]. Alterations in glycan structure can also occur by phase variation (the high frequency, reversible ON/OFF switching of gene expression) of the glycosyltransferases [10]. This potential diversity in the presence of glycosyltransferases leads to a corresponding diversity of potential mature glycan structures that can be transferred to protein. However, independent of the structure of the mature glycan, it is flipped to the periplasmic face of the inner membrane by PglF and then transferred to protein by the PglL *O*-oligosaccharyltransferase (*O*-OTase) [12]. This *O*-OTase enzyme exhibits extreme glycan substrate promiscuity, and is capable of transferring many structurally unrelated substrates from a pyrophosphate-polyprenyl carrier to protein, including the possible naturally occurring *N. meningitidis* glycans, *C. jejuni* glycan and even peptidoglycan subunits [13]. This promiscuity is presumably advantageous to allow efficient transfer of the diverse naturally occurring *Neisserial* glycans to facilitate immune evasion.

While the PglL *O*-OTase exhibits extreme glycan substrate tolerance, its acceptor protein range is not so diverse. Although *N. meningitidis* PglL and its homolog in *N. gonorrhoeae* are general *O*-OTases capable of glycosylating multiple substrate proteins, the range of substrate proteins is limited. *N. meningitidis* has only two reported glycoproteins, PilE and AniA [3]. PilE is an abundant glycoprotein adhesin of *N. meningitidis*, and is glycosylated on Ser63. This Ser is located in a folded domain with local sequence Asn-Thr-Ser(glycan)-Ala-Gly. A second glycoprotein, AniA is glycosylated in its low-complexity C-terminal region with up to two Ser residues modified [3].

The key difference between *wzy*-dependent O-antigen biosynthesis and PglL protein O-glycosylation is the acceptor substrate specificity: oligosaccharide is transferred by the WaaL O-antigen ligase to a terminal sugar of the LPS core, while the PglL *O*-OTase transfers oligosaccharide to serine or threonine in a protein. While WaaL and PglL catalyze reactions with clearly different biological roles, it is not possible to differentiate the two groups of enzymes using simple sequence based analyses such as BLAST [12].

Here, we describe the bioinformatic analysis of the PglL *O*-OTase and the systematic identification of PglL homologs in other bacterial species. We further characterized the protein acceptor substrate requirements of *N. meningitidis*, which together provide insights into the characteristics of general *O*-glycosylation systems in bacteria.

## Materials and Methods

### Identification and Alignment of Homologues of PglL

The BLASTP programme was used to examine the NCBI non-redundant protein database (http://www.ncbi.nlm.nih.gov/BLAST/) for homologs of the protein glycosylation ligase of *N. meningitidis* (PglL, NMA0800). 22 homologs, that were unique protein sequences from each genus, were downloaded (PIDs 15676501, 34499662, 153886317, 76809217, 50083393, 153886318, 126665137, 121611683, 91790502, 126643191, 124265256, 120613309, 86147861, 89900058, 148977877, 121609153, 120609538, 89075308, 119944149, 15891610, 150377063, 51245303, 110807324, 77958742, 145298462). ClustalW alignment of these proteins identified two regions that were well conserved in the putative PglL proteins and not in the WaaL proteins (O-antigen ligases). The first region, termed PglL_A, was 25 amino acids (equivalent to amino acid 395 to 420 of NMA0800) and the second, termed PglL_B, was 30 amino acids (equivalent to amino acid 171 to 201 of NMA0800). A HMM was generated from a ClustalW alignment of both of these regions in the above proteins using the program hmmbuild, followed by hmmcalibrate [14]. Membrane spanning regions of proteins were predicted using Phobius V 1.0 (http://phobius.sbc.su.se/) [15]. The graphical representation of the Phobius model was created with TMRPres2d [16].

### Bacterial Strains and Culture Conditions

*Acinetobacter baylyi* strain ADP1 and *Escherichia coli* strain DH5α, used to propagate cloned plasmids, were grown at 37°C in LB broth or on LB solid agar. *N. meningitidis* strains were grown on brain heart infusion medium (BHI) supplemented with Levinthal’s base. *N. gonorrhoeae* strains were grown on GC media with IsoVitaleX. Media was supplemented with appropriate antibiotics.

### DNA Isolation and Manipulation

Genomic DNA of *A. baylyi* strain ADP1 was used as template in a PCR reaction to amplify the *pglL* homolog (accession number ACIAD3337). The product was cloned into the pT7Blue vector, linearized with restriction endonuclease *Sty*I, blunted and ligated to the kan resistance cassette, excised from pUC4kan with *Hinc*II. Transformation of ADP1 was based on previously described methods [17]. Shuttle vector pWH1266 [18] was used to complement the ADP1*pglL::kan* mutant. The wild type ADP1 *pglL* gene including 61 bp of the upstream region was amplified by PCR, digested by BamHI and inserted into vector pWH1266 at the BamHI site. The resulting plasmid, pWH1266-*pglL,* and pWH1266 were separately transformed into the ADP1*pglL::kan* mutant by electroporation. Previously described plasmid encoding FLAG-tagged AniA and TetMB [3] was used as template for site-directed mutagenesis to construct AniA variants [19]. Linearized plasmid was transformed to the chromosome of C311 by homologous recombination as described [3]. *N. gonorrhoeae* MS11*pglL::Kan* strain was constructed as described [12].

### Immunological Assays

Western blotting was performed essentially as previously described [9]. Primary antibodies used were rabbit α-trisaccharide sera [9] and mouse α-FLAG mAb (Sigma-Aldrich). α-ComP (*A. baylvi*), and α-CcoP, α-MetQ, α-Sco, α-Mip and α-Laz (*N. meningitidis*) antibodies were produced by inoculating mice with the peptide-conjugated Keyhole Limpet Hemocyanin (synthesized by Mimotopes, Australia). The conjugated peptide sequences corresponding to the target proteins are shown in Table S1. Secondary antibodies used were anti-rabbit IgG and anti-mouse IgG (Sigma-Aldrich and Rockland). Cell lysates of wild-type and mutant strains of *A. baylyi* were prepared from cells in late stationary growth phase, when ComP expression is maximal [20].

### Protein Immunoprecipitation and Purification

*N. meningitidis* C311 cells were harvested and resuspended in TBSt (Tris buffered saline with 0.05% Tween-20) supplemented with protease inhibitor cocktail (Roche). Cells were heat killed by incubation at 56°C for 1 h, lysed by French press and debris removed by centrifugation at 18,000 rcf for 10 min and filtration through 0.22 µm filters. α-Glycan dynabeads for immunoprecipitation were prepared using rabbit polyclonal sera against the *N. meningitidis* C311 *O*-glycan [9] or isotype negative control antisera, and ProtA dynabeads (Invitrogen) according to the manufacturers instructions. Clarified cell lysate was applied to the α-glycan dynabeads and incubated with shaking at 25°C for 1 h. The α-glycan dynabeads were washed thrice with 1 mL TBSt and eluted with 200 µL (Glycine HCl pH 3 with 0.1% Tween-20). FLAG-tagged AniA proteins were purified as described [3].

### Mass Spectrometry

Purified AniA-FLAG protein was precipitated by addition of 4 volumes of 1∶1 acetone:methanol, incubation at −20°C for 16 h and centrifugation at 18,000 rcf for 10 min. The pellet was washed with acetone:methanol, dried, resuspended in 50 µL 50 mM NH_4_HCO_3_ with 1 µg trypsin (proteomics grade, Sigma) and digested at 37°C for 3 h. Peptides and glycopeptides were analysed by LC-ESI-MS/MS with an API QSTAR Pulsar i LC/MS/MS system, and MS data was analysed as described [3]. Differences in glycosylation occupancy between AniA variant proteins were compared using a 2-sided Mann-Whitney test. Immunoprecipitated eluted proteins were reduced/alkylated and digested as above. Peptides were desalted with C18 ZipTips (Millipore) and analysed by LC-ESI-MS/MS using a nanoLC (Shimadzu) and TripleTof 5600 mass spectrometer (ABSciex) as described [21]. Peptides were separated on a C18 column (VYDAC), with a gradient from buffer A (0.1% formic acid) to buffer B (80% acetonitrile with 0.1% formic acid)**.** Data was exported from.wiff format to.mgf format, and searched with MASCOT V2.3 at the Australian Proteomics Computational Facility (http://www.apcf.edu.au/). Search parameters were: enzyme, trypsin with up to 1 missed cleavage; fixed modifications, cysteine propionamide; variable modifications, methionine oxidation and asparagine deamidation; peptide tolerance, 0.05 Da; MS/MS tolerance, 50 mmu; LudwigNR database (as at 2 November 2011; 15,321,871 sequences; 5,325,977,554 residues) limited to *N. meningitidis* and common contaminants (28,591 sequences).

### Circular Dichroism Spectroscopy

CD spectroscopy of synthesized peptide (NGAAPAASAPAASAPAASASEKSVY; Auspep) in 50 mM potassium phosphate buffer at pH 6.5 buffer was performed using a Jasco J-710 spectrometer as described [22]. The data were collected in the wavelength range 190–269 nm at room temperature. The scan speed was set to be 100 nm/min and the bandwidth was 0.5 nm. Spectra were also obtained from solutions that contained the peptide in 10%, 20%, 30%, 40% and 50% trifluoroethanol (TFE).

### Molecular Modelling

Modelling of the amino acid sequence corresponding to the glycosylated region (^57^WPGNNTS (Gal(β1–4)Gal(α1–3)2,4-diacetimido-2,4,6-trideoxyhexose) AGVASSSTIK^73^) of C311#3 pilin was calculated and modelled by Chemdraw and DYANA. The two ends of the peptide (W57 and K73) were constrained 19.7 Å apart as in the corresponding region of *N. gonorrhoeae* pilin according to its published pili crystal structure [23].

## Results

### Creation of Hidden Markov Models to Distinguish between PglL and WaaL Candidates

**Figure 1:**
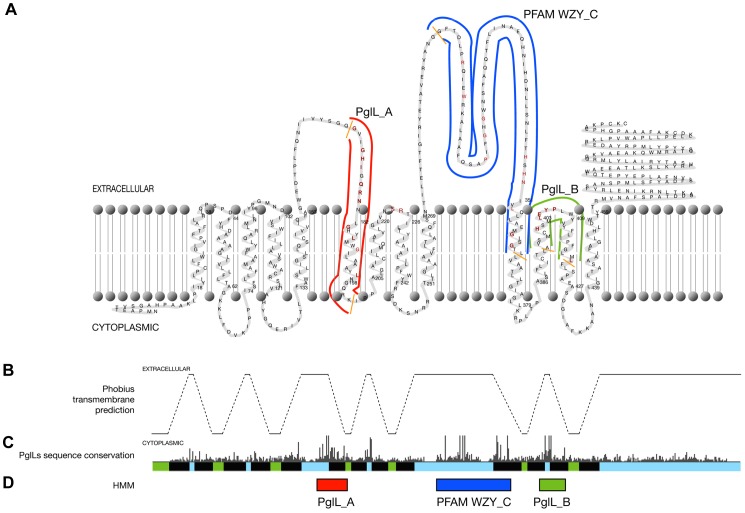
*N.meningitidis* PglL topology and conservation. (**A**) Transmembrane profile of *N. meningitidis* PglL with the regions identified by the PglL_A, PglL_B and Wzy_C hidden Markov models indicated by red, green and blue lines and highly conserved amino acids coloured in red and orange. (**B**) The PglL protein Phobius transmembrane helix prediction with predicted transmembrane regions represented by dashed-lines. (**C**) CLUSTALX plot of sequence conservation of CLUSTALW alignment of the putative PglL proteins. (**D**) Regions identified by the PglL_A, PglL_B and Wzy_C hidden Markov models indicated by red, green and blue boxes respectively.

To identify PglL homologs in bacterial genomes we developed a hidden Markov model (HMM) that would resolve the subset of PglL protein *O*-OTases from the wider PFAM PF04932, which contains both WaaL O-antigen ligases and PglL proteins. This family of enzymes has low overall amino acid similarity but contains a small region of conservation that is the basis for PFAM PF04932. To identify sequence features which accounted for the protein acceptor substrate specificity of PglL, we performed multi-sequence alignments of protein sequences of close homologues of PglL and used conserved features not present in WaaL to create two HMMs, pglL_A and pglL_B (Fig. 1). HMM pgl_A has been submitted to the Pfam database with accession number PF15864. These HMMs did not identify well-characterized WaaL proteins from enteric organisms, suggesting that they may be useful for the identification of PglL candidates in wider searches.

### Identification of PglL Homologs in Bacterial Genomes

The pglL-A HMM was used to search the NCBI non-redundant protein database to identify candidate PglL *O*-OTases (Table 1). Similar results were obtained with the pglL_B search. This analysis identified PglL homologs in 31 distinct Gram-negative bacterial species. These included pathogens such as *Burkholderia, Vibrio, Yersinia, Aeromonas* and *Acinetobacter,* and several non-pathogenic environmental species including *Polaromonas, Rhodoferax* and *Methylibium.*

**Table 1:** Proteins matching HMMs pglL_A with a score greater than 20.

PID:	Score	E value	Species	Genetic context*
91790502	51.6	1.60E-09	*Polaromonas sp. JS666*	pilin, 2
89900058	49.3	7.90E-09	*Rhodoferax ferrireducens T118*	pilin
126643191	48.6	1.30E-08	*Acinetobacter baumannii ATCC 17978*	pilin
120613309	47.7	2.40E-08	*Acidovorax avenae subsp. citrulli AAC00-1*	pilin
121611683	47.3	3.20E-08	*Verminephrobacter eiseniae EF01-2*	pilin
84394373	47	3.90E-08	*Vibrio splendidus 12B01*	EA
148977877	47	3.90E-08	*Vibrionales bacterium SWAT-3*	EA
50083393	46.7	4.90E-08	*Acinetobacter sp. ADP1*	GalE, Pmm
59800633	46.3	6.40E-08	*Neisseria gonorrhoeae FA 1090*	hypothetical membrane protein
15793774	46.3	6.40E-08	*Neisseria meningitidis*	hypothetical membrane protein
124265256	45.9	8.30E-08	*Methylibium petroleiphilum PM1*	pilin
119944149	45.8	9.20E-08	*Psychromonas ingrahamii 37*	E, transglycosylase SLT domain protein
126665137	44.9	1.70E-07	*Marinobacter sp. ELB17*	pilin
86147861	44.6	2.10E-07	*Vibrio*	EA
121609153	43.6	4.30E-07	*Verminephrobacter eiseniae EF01-2*	acyltransferase 3, ribonuclease II
21672281	42.7	7.90E-07	*Aeromonas hydrophila*	wzz
121532578	42.3	1.00E-06	*Ralstonia pickettii 12J*	pilin
90412963	40.2	4.30E-06	*Photobacterium profundum 3TCK*	EA
17545278	39.6	6.80E-06	*Ralstonia solanacearum*	pilin
51245303	39.5	7.20E-06	*Desulfotalea psychrophila LSv54*	pilin
77979651	39.2	8.60E-06	*Yersinia intermedia ATCC 29909*	pilin
134279817	38.3	1.60E-05	*Burkholderia pseudomallei 305*	pilin
53724725	38.3	1.60E-05	*Burkholderia mallei ATCC 23344*	pilin
76809217	38.3	1.60E-05	*Burkholderia thailandensis E264*	pilin
153886317	38.2	1.80E-05	*Ralstonia pickettii 12D*	pilin, 2
34499662	38	1.90E-05	*Chromobacterium violaceum ATCC 12472*	pilin, 2
149911011	37.8	2.30E-05	*Moritella sp. PE36*	EA
123441130	36.6	5.30E-05	*Yersinia enterocolitica 8081*	pilin
77958742	36.6	5.30E-05	*Yersinia bercovieri ATCC 43970*	pilin
91790501	36.2	6.80E-05	*Polaromonas sp. JS666*	pilin, 2
121595941	35.9	8.40E-05	*Acidovorax sp. JS42*	cytochrome c
90407310	35.9	8.80E-05	*Psychromonas sp. CNPT3*	EA
121606830	35.8	9.50E-05	*Polaromonas naphthalenivorans CJ2*	pilin

* Genetic context: EA, *pglL* between homologues of a exonuclease ABC subunit A and aminotransferase class V; pilin, a type IV pilin homologue, pilin 2, two closely associated pilin homologues; E, exonuclease ABC subunit A; other genes are described by their homology or commonly known gene names.

**Figure 2:**
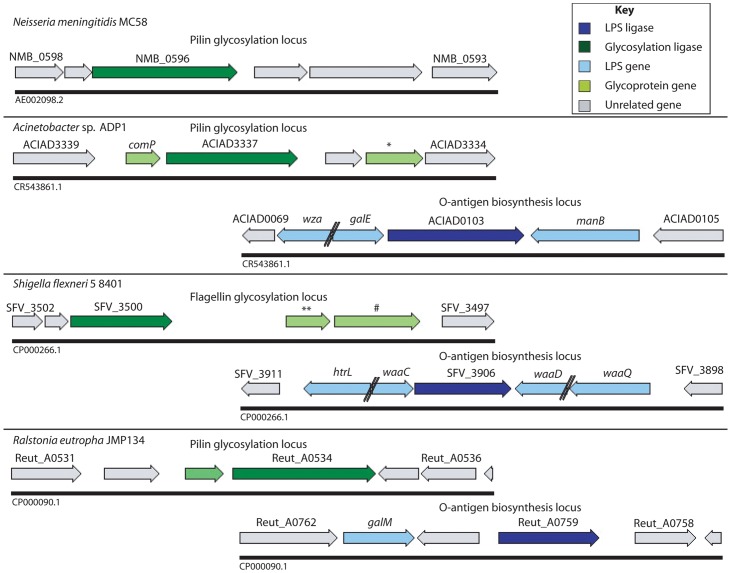
Genomic arrangement of selected *pglL* genes. Schematic representation of the regions of the genome containing *pglL* genes in *Neisseria meningitidis*, *Acinetobacter sp.*, *Shigella flexneri* and *Ralstonia eutropha*.

Examination of the genome location of these *pglL* homologs revealed that in the majority of cases (21/31) they were immediately adjacent to or closely associated with a gene(s) encoding type IV pilin homologs (Table 1, Fig. 2). The close association of the *pglL O*-OTase with an obvious target glycoprotein in so many cases suggests that the HMM analysis identified both the glycosylation pathway and target acceptor protein. A further indication that the genes identified are PglL rather than WaaL homologs is that they are not located within LPS biosynthetic loci [24].

The PglL_A and PglL_B motifs are located on either side of the Wzy_C motif common to both PglL and WaaL, and represent conserved regions on predicted periplasmic loops of PglL_Nm_ and adjacent transmembrane regions. This suggests they have an important structural or functional role in PglL activity. Three residues important for the function of *E. coli* WaaL [25] located in the Wzy_C motif are also conserved amongst PglLs (*E. coli* WaaL R288, H338 and R215; *N. meningitidis* PglL R298, H349, R224). We also identified additional residues conserved in PglLs but not in WaaL (*N. meningitidis* PglL Q178, N180, G316, G318, H400, E404, P406) and residues conserved in both PglLs and WaaL (*N. meningitidis PglL* P313).

### Experimental Confirmation of O-OTase Activity in a pglL Homolog from Acinetobacter Baylyi

**Figure 3:**
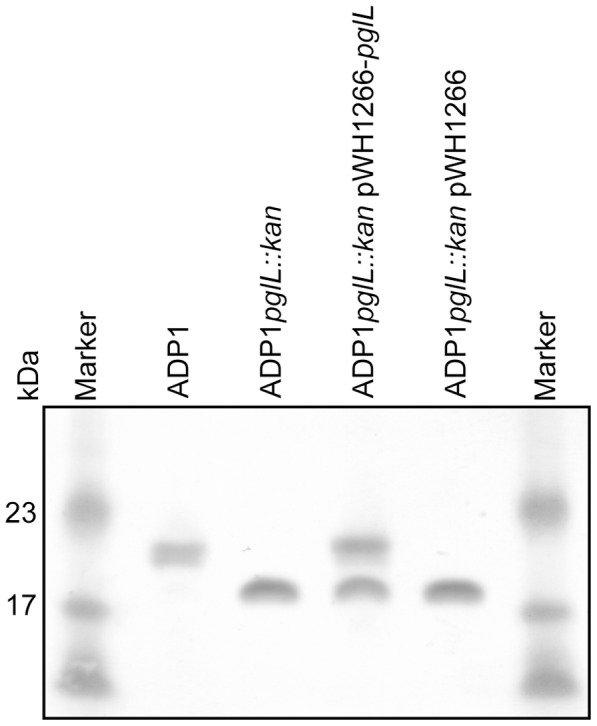
Phenotypic analysis of *Acinetobacter baylyi* strain ADP1 candidate PglL O-OTase mutants. Western blot analysis using α-ComP antibody of wild-type (ADP1), *pglL* mutant (ADP1*pglL::kan*), *pglL* mutant complemented with plasmid-borne *pglL* (ADP1*pglL::kan* pWH1266-*pglL*) and *pglL* mutant with empty vector (ADP1*pglL::kan* pWH1266).

We tested the hypothesis that the pilin homologs closely associated with the PglL *O*-OTase candidates were the cognate target glycoproteins. In *A. baylyi* strain ADP1, the *pglL* gene (accession number ACIAD3337) is adjacent to the *comP* gene (ACIAD3338) which encodes a pilin-like protein which is essential for natural transformation and has previously been shown to be glycosylated [20]. However, the mechanism of glycosylation of ComP has not previously been investigated. We created a knockout mutant in the *pglL* gene of *A. baylyi* strain ADP1, and complemented this mutant strain with expression of plasmid-borne native *pglL*. Western blot analysis of extracts from the wild-type ADP1 and ADP1*pglL::kan* mutant strains using an α-ComP antibody indicated the presence of the 20 kDa glycosylated ComP protein in the wild-type strain and a shift in MW to the 18 kDa non-glycosylated form of ComP in the mutant strain (Fig. 3), consistent with the loss of glycosylation of this protein. This glycosylation could be partially rescued by complementation with native *pglL*, but not with empty vector. This validated the role of the PglL homolog in glycosylation of the ComP pilin-like protein in strain ADP1.

### General PglL Glycosylation Systems

The genomic localization of *pglL* close to substrate glycoproteins in most bacteria suggested that these substrates were the key targets for glycosylation (Table 1, Fig. 2). However, several *pglL* homologs were detected not genomically associated with an obvious glycoprotein substrate, including in the pathogenic *Neisseria* known to possess general glycosylation systems. Since genes that are not genomically linked cannot be co-transcribed, we anticipated that additional mechanisms based on enzyme-substrate recognition would also enhance glycosylation efficiency in these bacteria. We therefore used *N. meningitidis* as a model system to investigate the substrate requirements for modification in this genomically unlinked system.

**Figure 4:**
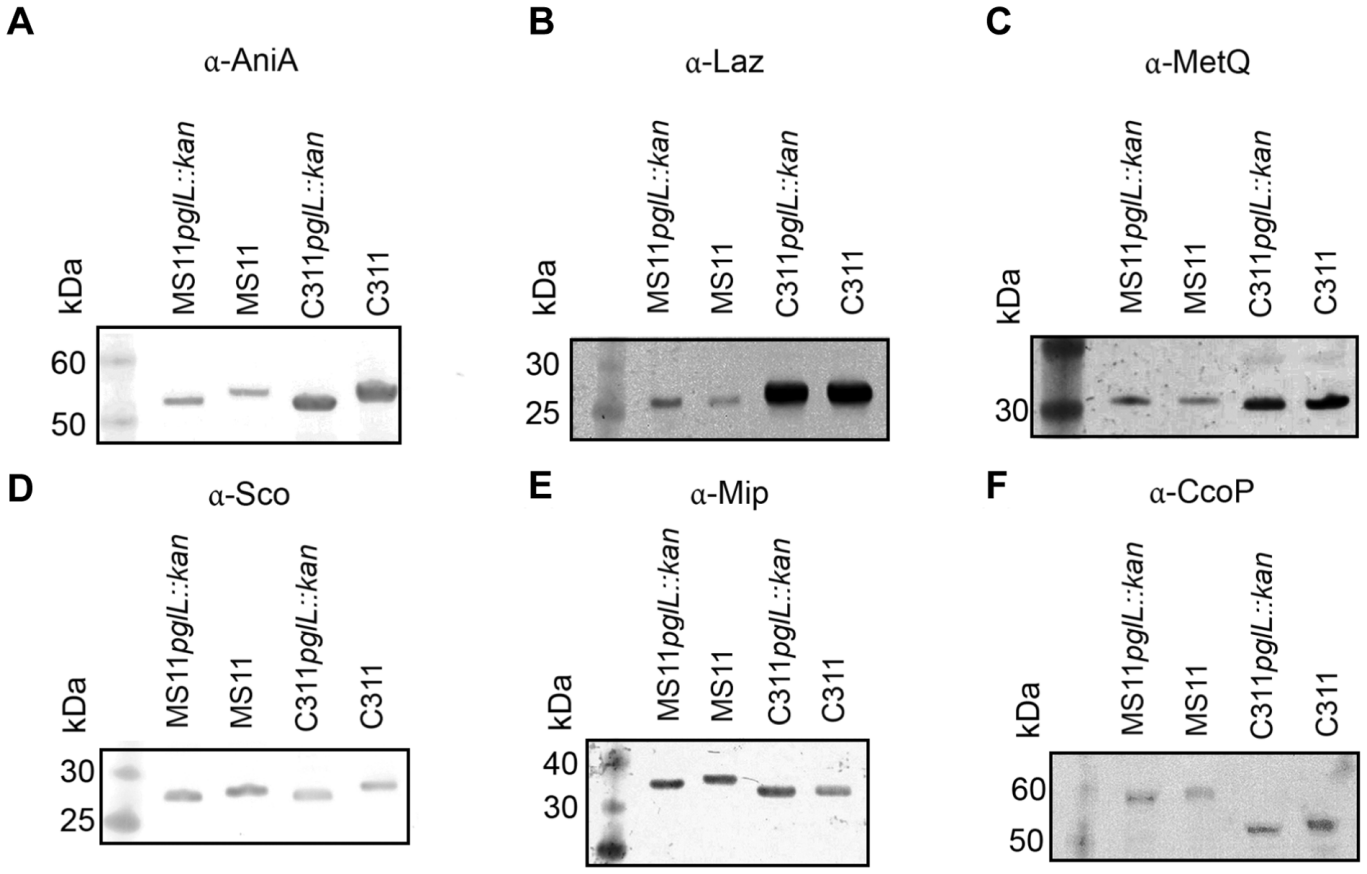
Western blot analysis of putative *Neisserial* glycoproteins. *N. gonorrhoeae* MS11*pglL::kan*, *N. gonorrhoeae* MS11, *N. meningitidis* C311*pglL::kan* and *N. meningitidis* C311 whole cell extracts were separated by SDS-PAGE, blotted to nitrocellulose membrane and probed with (**A**) α-AniA, (**B**) α-Laz, (**C**) α-MetQ, (**D**) α-Sco, (**E**) α-Mip or (**F**) α-CcoP antisera.

Several PglL substrate glycoproteins in addition to PilE and AniA have been reported in *N. gonorrhoeae* [4]. However, Western blotting using our α-glycan antisera failed to detect bands in addition to AniA and PilE in *N. meningitidis* C311 whole cell extracts [3]. To investigate if other glycoproteins were also present in *N. meningitidis* C311, we performed IP of whole cell extracts using α-glycan antisera, and identified eluted proteins with mass spectrometry. α-Glycan co-IP identified three proteins: PilE, azurin and MetQ (Fig. S1, S2 and S3; Tables S2, S3, S4 and S5). These proteins were not identified by negative control IP with unrelated rabbit antisera, suggesting that they were glycoproteins. To validate the glycosylation status in *N. meningitidis* of azurin and MetQ, as well as selected other reported *N. gonorrhoeae* glycoproteins [4], we performed western blotting with protein-specific antisera for each candidate glycoprotein. This showed that AniA, Sco, CcoP and Mip were glycosylated in *N. meningitidis* C311 and *N. gonorrhoeae* MS11, as they displayed clear MW shifts upon genomic deletion of the PglL O-OTase (Fig. 4A,D,E,F). However, MetQ and Laz failed to show clear changes in MW in the presence and absence of glycosylation (Fig. 4B,C). This phenotype was also observed in *N. meningitidis* MC58, and in *N. gonorrhoeae* 1291 and O1G1370. Homologs of all of these proteins had been identified as glycoproteins in *N. gonorrhoeae* [4]. Together with our α-glycan IP results, this suggests that although glycosylated forms of MetQ and Laz can be detected by MS [4], the major fraction of these proteins in the cell under the conditions tested is not in fact glycosylated.

### Characteristics of Efficiently Glycosylated PglL Protein Acceptor Substrates

The protein substrates of *N. meningiditis* PglL were either predominantly glycosylated or predominantly non-glycosylated. PilE, AniA, Sco, CcoP and Mip were completely glycosylated, as their non-glycosylated forms were not detectable by western blot in wild type *Neisseria*. Laz and MetQ were minimally glycosylated, as their glycosylated forms were not detectable by western blot, but rather only by MS analysis after glycan-specific enrichment. To determine the details of this substrate specificity, we examined the *N. meningitidis* glycoprotein AniA, which has been shown to be glycosylated with up to two glycans in its C-terminal flexible domain within the glycopeptide L_358_SDTAYAGNGAAPAASAPAASAPAASASEK_387_ [3]. No additional sites of glycosylation were detected by this MS analysis [3]. We first tested if AniA had additional glycosylation sites by analysing a FLAG-tagged AniA variant with this 36 amino acid C-terminal flexible domain deleted after Met354. Western blot analysis of FLAG-tagged full-length wild type and C-terminally truncated variant AniA using α–FLAG sera detected both variants, but α–glycan sera only detected full length AniA (Fig. S4). This indicated that no additional glycosylation sites were present in the core nitrite reductase or flexible N-terminal domains of AniA.

**Figure 5:**
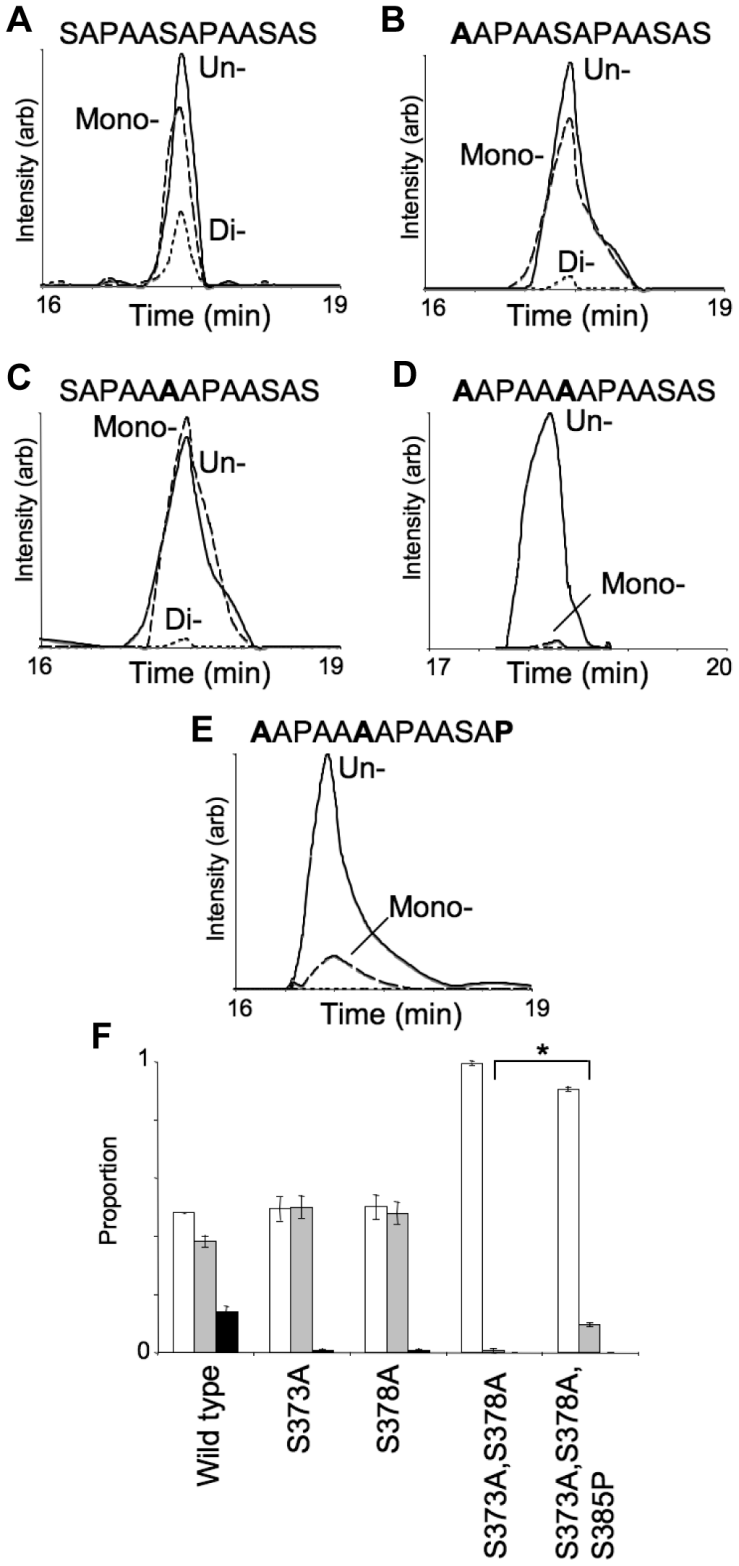
Glycosylation status of purified AniA-FLAG variants. Extracted ion chromatograms corresponding to the un- (full) mono- (dashed) and di- (dotted) glycosylated versions of the Leu358-Lys387 tryptic peptide containing the AniA glycosylation sites: (**A**) wild type, (**B**) S373A, (**C**) S378A, (**D**) S373A, S378A, (**E**) S373A, S378A, S385P. Corresponding variant sequences are shown in Table S6. (**F**) Proportion of un- (white) mono- (gray) or di- (black) glycosylated versions of each variant shown in (A)–(E), as determined by integration of extracted ion chromatograms. Values are mean, error bars show s.e.m. *, *P* = 0.01 2-sided Mann-Whitney test.

We identified the precise sites of glycosylation in the Leu358-Lys387 glycopeptide by site-directed mutagenesis and LC-ESI-MS/MS analysis of peptides and glycopeptides from purified variant glycoproteins. The extent of glycosylation is likely under-estimated by this MS analysis due to reduced ionisation efficiency of the glycosylated peptides relative to their unglycosylated forms. Nonetheless, relative quantification of glycosylation occupancy is possible with this analysis [26], [27]. Up to two sites of glycosylation were detected in wild type AniA (Fig. 5A, Fig. S4 and [3]). As this sequence included two identical repeats of the local sequence Ala-Ala-Ser-Ala-Pro, encompassing Ser373 and Ser378, we created AniA variants with each of these Ser residues individually mutated to Ala (Table S6). LC-ESI-MS/MS analysis of both of these variants showed loss of a single efficiently modified glycosylation site, as these variants showed very low levels of di-glycosylated peptide (Fig. 5B,C,F). Further, a variant with both Ser373Ala and Ser378Ala mutations showed loss of both efficiently used glycosylation sites, as essentially only un-glycosylated peptide was identified (Fig. 5D,F). This confirmed that the two Ser residues present in the local sequence Ala-Ala-Ser-Ala-Pro (Ser373 and Ser378) were efficiently glycosylated by PglL. Several other Ser residues are present in the Leu_358_-Lys_387_ glycopeptide, and of particular note were the residues present in an imperfect repeat reminiscent of the efficiently glycosylated sites, Ala-Ala-Ser383-Ala-Ser385-Glu. The local sequence context of Ser383 differed from the efficiently glycosylated Ser373 and Ser378 only by having the sequence Ser383-Ala-Ser rather than Ser373/8-Ala-Pro. We tested if this local sequence influenced glycosylation by creating a Ser385Pro AniA variant in a Ser373Ala, Ser378Ala background. Indeed, this AniA-Ser373Ala, Ser378Ala, Ser385Pro variant showed significantly increased glycosylation compared with the Ser373Ala, Ser378Ala control, with substantial mono-glycosylated peptide detected (2-sided Mann-Whitney test, *P* = 0.01, Fig. 5E,F).

**Figure 6:**
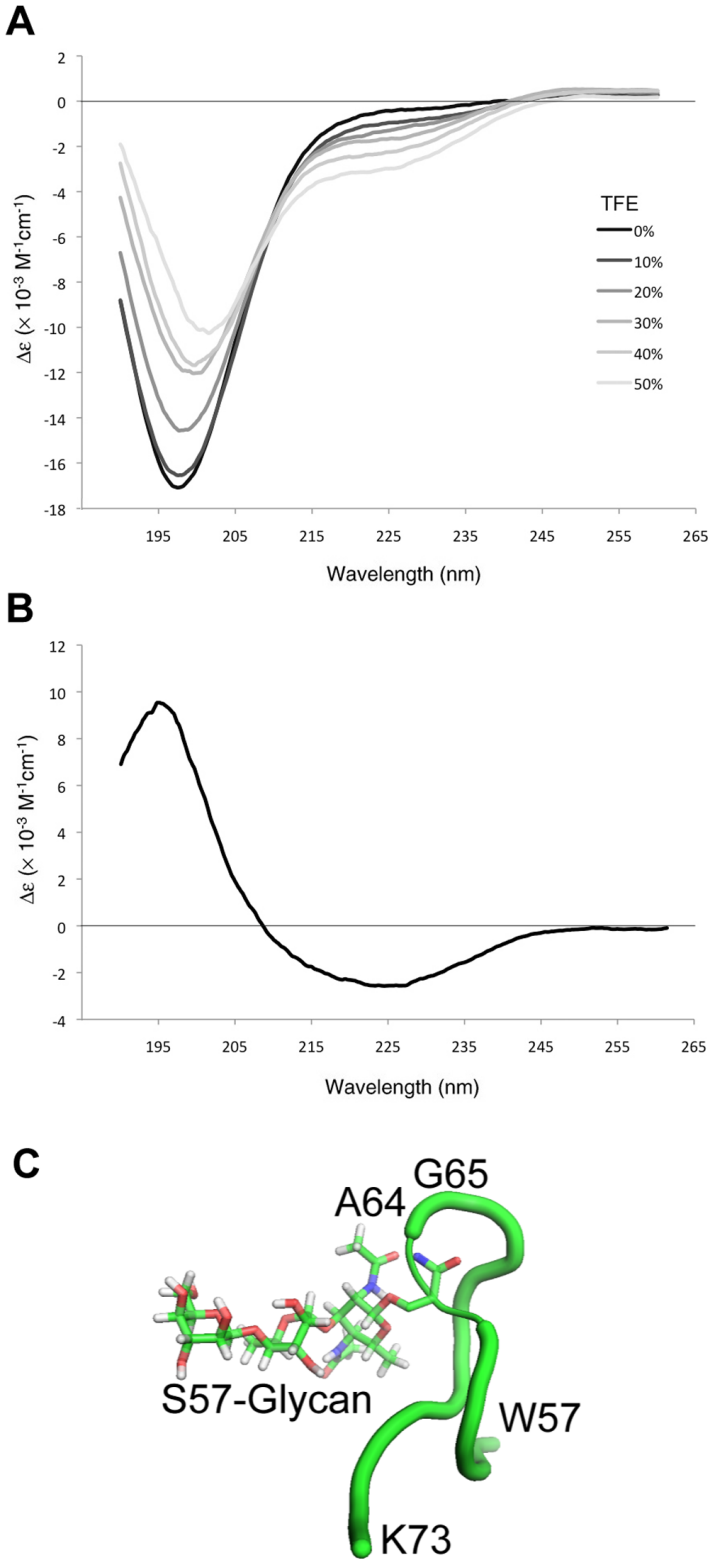
Structures of PglL acceptor substrate proteins. (**A**) CD Spectrum of AniA C-terminal peptide (NGAAPAASAPAASAPAASASEKSVY). Peptide was analysed in 50 mM KH_2_PO_4_ with 0–50% of TFE. (**B**) The difference in CD spectra between the peptide in 50% TFE and no TFE. (**C**) Modelling of *N. meningitidis* PilE glycosylation site structure. Peptide corresponding to the glycosylated region of C311 PilE (^57^WPGNNTS(Gal(β1–4)Gal(α1–3)2,4-diacetimido-2,4,6-trideoxyhexose)AGVASSSTIK^73^) constrained as in the structure of *N. gonorrhoeae* PilE was modelled.

To investigate the structure of the AniA glycosylation acceptor sites, we performed circular dichroism (CD) spectroscopy to characterize the secondary structure of a synthesized peptide corresponding to the unglycosylated AniA glycopeptide. The CD spectrum of AniA in phosphate buffer showed substantial negative ellipticity centred at 198 nm (Fig. 6A), indicative of an unstructured conformation. The glycosylation site in PilE (NTS_63_(glycan)AG) is part of a short α-helix located in the so called ‘ab loop’ [23], so to investigate if the AniA peptide in solution samples an energy landscape that contains transiently structured conformations we obtained CD spectra in the presence of increasing concentrations of TFE, which allows peptide intramolecular hydrogen bonds to form by limiting competing bond formation with solvent water. The CD spectra showed that increasing TFE caused loss of negative ellipticity at 198 nm with a corresponding increase in negative ellipticity around 225 nm (Fig. 6). Subtraction of the spectrum of the AniA peptide in 0% TFE from that in 50% TFE resulted in a spectrum with positive ellipticity at 195 nm and a broad negative peak centred on 220 nm (Fig. 6B). These features are suggestive of a helical conformation, but we note that even in 50% TFE the AniA peptide was still predominantly unstructured. NMR analysis (Fig. S5) of the AniA peptide in the absence of TFE showed that most amide protons had chemical shifts clustered between 8.1 ppm and 8.3 ppm; Val24 and Tyr25 were shifted upfield due to Tyr ring current effects. In agreement with the CD results, the NMR result was indicative of an unstructured conformation. The presence of increasing concentrations of TFE showed a corresponding increase in the dispersion of amide chemical shifts (both upfield and downfield shifts were observed) suggesting that some residues adopted a structured conformation.

## Discussion

Post-translational modifications of bacterial proteins are difficult to identify and a bioinformatic means of identifying potential glycosylation systems and their targets would enable the characterisation of many more systems. PglL proteins in particular have been difficult to identify due to the relatively low levels of homology between members of this family and the overlap in similarity to WaaL O-antigen ligases. Previously, PglL *O*-OTases have been identified by homology with known WaaL/*O*-OTases and the presence of the Wzy_C motif common to these distinct functions [12], and then *O*-OTase function distinguished from WaaL O-antigen ligase function by experimentation using mutagenesis of the putative gene or cloning and expression in a recombinant system [28]. In the current study we identified two conserved motifs (PglL_A and PglL B) that are found in PglL homologs but not in WaaL O-antigen ligases (Fig. 1). *In silico* analysis using these motifs identified *pglL* genes in diverse Gram-negative bacteria, showing that bacterial protein glycosylation systems are much more common than previously appreciated. The PglL homolog we identified from *Y. enterocolitica* (Table 1) (Ye777; *waaL_XS_*; protein accession 123441130) has been studied in *Y. enterocolitica* and in *E. coli* for a role in LPS biosynthesis. *Y. enterocolitica* encodes three WaaL homologs, and while all three could complement LPS biosynthesis with deletion of *E. coli waaL*, Ye777/*waaL_XS_* was not involved in LPS biosynthesis in the native organism *Y. entercolitica* [29]. This is consistent with Ye777/*waaL_XS_* being a protein *O*-OTase, as previously speculated [29] and as predicted by our HMM analysis. During the preparation of this manuscript, PglL-like *O*-OTase BTH_I0650 of *B. thailandensis* [30], VC0393 of *V. cholerae* [30] and A1S_3176 of *A. baumannii* [31] were identified by homology with *Neisseria* PglL and the presence of the Wzy_C motif followed by experimentation to rule out a WaaL function. Our HMM analysis predicted that these genes were PglL homologs (Table 1), providing further support for our analysis. The bioinformatic approach described herein efficiently predicts many other such protein *O*-glycosylation systems.

In *P. aeruginosa*, the PilO system glycosylates pilin via the addition of a single unit of the LPS O-antigen repeat [32]. The *pilO* gene is adjacent to the pilin gene and in the same orientation, however, the PilO O-OTase, despite sharing similarity with PglL of *Neisseria* and O-antigen ligases, does not contain the PglL motifs we describe here. Indeed, examination of protein databases does not reveal homologs with a high degree of similarity to PilO outside of the *P. aeruginosa* species suggesting that PilO might have a slightly different mode of action to the PglL family.

The *pglL* gene and the gene encoding its presumptive target protein are often found in the same orientation and in close proximity, which suggests they are co-transcribed. This may increase protein co-localisation and thereby increase the efficiency of the glycosylation reaction. However, the requirement of genomic co-localisation is not absolutely required. PglL of *Neisseria* is not located adjacent to the pilin structural gene, *pilE*, and *N. meningitidis* and *N. gonorrhoeae* have general *O*-glycosylation systems capable of modifying serine and threonine residues in many different protein substrates [3], [4]. Apart from PilE, which is the most abundant glycoprotein, the additional substrates are modified in flexible, low-complexity regions [3], [4]. However, the factors controlling selection of particular sites for glycosylation in these domains are poorly understood. Our results here show that the local sequence Ala-Ala-Ser-Ala-Pro found in AniA, in particular the Pro, allows efficient glycosylation of Ser residues (Fig. 5). However, in all of the variant proteins examined, extremely low levels (<1%) of glycosylation at additional sites were repeatedly detected (Fig. 5). This suggested that PglL does not recognize a strictly defined ‘sequon’, but rather glycosylates Ser residues with different efficiencies or rates depending on the local sequence or structural environment. Comparison of the AniA and PilE glycosylation sites reveals that only the primary amino acid sequence Ser(glycan)-Ala is common. This sequence is unlikely to define the PglL substrate, as the same sequence occurs in many other non-glycosylated proteins, and also in other, non-glycosylated regions of AniA and PilE. The pilin target serine is in a surface loop, on a section that contains a short α-helix (Fig. 6C, and [23]). Secondary structure may therefore be a key aspect of PglL substrate specificity. This is supported by our *in vitro* CD spectroscopy and *in vivo* mutagenesis analysis of AniA glycosylation acceptor sites. CD spectroscopy of the AniA peptide in solution with TFE suggested that this peptide samples conformations that contain regular helical structures (Fig. 6). Further, the proline residues proximal to glycosylated serines in AniA (Fig. 5) may temporarily introduce the local structure required for efficient glycosylation. This may be similar to peptide substrate binding shown in the X-ray structure of the *Campylobacter lari N*-OTase [33], where the ‘N-glycosylation sequon’ (D/E-x-N-z-S/T) [5] is not required for catalysis, but rather for protein acceptor recognition. The requirement that *N. meningitidis* PglL substrates have a turn immediately C-terminal to the serine to be glycosylated may be due to specific interactions resulting in increased acceptor protein binding affinity, or spatial constraints at the *O*-OTase active site. This is consistent with this polypeptide sequence binding to PglL with an induced fit mechanism, or that the proline adjacent to the target sites key for glycosylation in AniA may give rise to local temporary turns mimicking the constrained loop in PilE.

It has been shown that purified PglL protein can be used in *in vitro* assays to glycosylate substrate protein in the presence of solubilized glycan-pyrophosphate-undecaprenyl as donor glycan substrate [34]. In these *in vitro* assays, the *N. gonorrhoeae O*-OTase could glycosylate purified PilE protein, but could not glycosylate a short peptide containing the glycosylation site and surrounding residues. This is in agreement with our data, consistent with local structural conformation rather than sequence being key to recognition by the PglL *O*-OTase. Interestingly, an unrelated class of bacterial cytoplasmic *O*-glycosyltransferases also recognize a structural motif in its protein acceptor substrates [35]. In all *Neisserial* glycoproteins except for PilE, glycosylation occurs in flexible linker extensions that are N- or C-terminal or linking two domains [4]. Such extensions would be likely to minimally impact protein folding and function, and as such would not have large evolutionary barriers. It is likely that these factors have allowed the *Neisserial* glycosylation system to evolve from an ancestral targeted system that modified the single abundant PilE substrate, to a general system with substrates that contain flexible N-, internal or C-terminal extensions that are local structural mimics of the PilE glycosylation site. Efficient glycosylation of only folded PilE by PglL [34] implies that glycosylation *in vivo* in the *Neisseria* most likely occurs only after protein folding. This is in contrast to the distantly homologous system of N-glycosylation by bacterial *N*-OTase, which glycosylates asparagines in unfolded polypeptide or flexible regions of folded proteins [36]. *N*-OTase is generally coupled to polypeptide translocation to access unfolded protein substrate [37], and this contrast suggests that PglL may have a different sub-compartmental localisation which allows efficient post-folding glycosylation of proteins including PilE, AniA, Sco, CcoP and Mip, but which allows only limited modification of other substrates including MetQ and Laz. Sco, CcoP and Mip are periplasmic, and as such may have prolonged access to PglL, allowing efficient glycosylation. In contrast, MetQ, Laz and AniA are outer membrane proteins, and so must be glycosylated by PglL before transport from the periplasm to the outer membrane. Efficient modification of AniA may be due to the C-terminal position of its flexible glycosylated domain, in contrast to the N-terminal flexible domains of MetQ and Laz. Additional factors including protein-specific secretion rate or subcellular targeting may also be important in controlling the efficiency of glycosylation of particular proteins. We note that previous reports characterising the glycosylation system of *N. gonorrhoeae* [4] relied on MS identification of enriched glycoproteins and an *ex vivo E. coli* expression system to identify PglL substrate proteins. As such, differences in efficiency of glycosylation were not distinguished. During the preparation of this manuscript seven additional putative glycoproteins were reported from *N. gonorrhoeae* using a glycan-specific enrichment and MS identification strategy [38]. Subsequent analysis of His-tagged versions of these putative glycoproteins expressed in *N. gonorrhoeae* revealed that many of these proteins were essentially unmodified, as we also observed in the current study (Fig. 4) for several other candidate glycoproteins reported by the authors in a previous study [4]. These stark differences in the glycosylation efficiency of the various putative *Neisserial* glycoproteins were not discussed by the authors [38]. However, their data and our current study emphasize the importance of studying the native organism using a range of complementary analytical techniques in determining if a protein is efficiently glycosylated, and thereby appropriately defined as a glycoprotein, rather than being a minor or accidental substrate only identified by very sensitive glycan-specific enrichment and MS detection.

The ability of the *O*-glycosylation system of the pathogenic *Neisseria* to modify many protein substrates may be related to the need to physically unlink the genes encoding PglL and the pilus biogenesis machinery. The *pilE* gene of *Neisseria* is highly antigenically variable and the system that promotes antigenic variation in *pilE*, by high levels of homologous recombination between *pilE* and non-expressed copies of the gene *pilS,* is dependent on the context of the *pilE* gene for efficient recombination between *pilE* and *pilS* [39]. There may therefore have been selective pressure for the *pglL* gene to be located distally from the *pilE* gene in *Neisseria*. The close genomic location of *pglL* and substrate glycoprotein in many bacteria likely confers efficiency to the glycosylation machinery. Non-linked *pglL* would therefore likely require increased protein acceptor substrate recognition or alternative substrate targeting mechanisms. Such features of efficient protein substrate selection, other than genomic location, could then allow the evolution of efficient glycosylation sites in many proteins. Unlike sequon-based recognition in general N-glycosylation systems [5], [36], [40], structural features dominate recognition of protein substrates in the general Neisserial *O*-linked glycosylation system.

## Supporting Information

Figure S1 Peptide mapping coverage of Azurin (NMB_1533) after IP with α-glycan antisera. Peptides identified with p<0.05 (ions score >23) are underlined.
(TIF)

Figure S2 Peptide mapping coverage of PilE (NMB_0018) after IP with α-glycan antisera. Peptides identified with p<0.05 (ions score >23) are underlined.
(TIF)

Figure S3 Peptide mapping coverage of MetQ (NMB_1946) after IP with α-glycan antisera. Peptides identified with p<0.05 (ions score >23) are underlined.
(TIF)

Figure S4 *N. meningitidis* AniA glycosylation (**A**) Cartoon of domains of *N. meningitidis* AniA protein showing: lipid-anchored N-terminal cysteine; N-terminal flexible region; AniA core fold; glycosylated C-terminal flexible region; Δ, truncated variant at Met354. (**B**) Western blots of FLAG-tagged purified AniA: wild type and C-terminally truncated (at Met 354, Δ in (A)), detected with either anti-FLAG or anti-glycan antisera.
(TIF)

Figure S5 750 MHz NMR spectra of the AniA glycosylation peptide in increasing concentrations of TFE-*d*6 (in 20 mM KPi, pH 6.5). The amide and aromatic region is shown (6.6–8.6 ppm). Increasing TFE-*d*6 results in upfield and downfield shifts of amide resonances. The shift of V389 and Y390 are shown dashed.
(TIF)

Table S1 Peptide sequences used for conjugate to Keyhole Limpet Hemocyanin to raise protein-specific antisera.
(PDF)

Table S2 Proteins identified from *N. meningitidis* after IP with α-glycan antisera.
(PDF)

Table S3 Peptides identified from Azurin (NMB_1533) after IP with α-glycan antisera with p<0.05 (ions score >23).
(PDF)

Table S4 Peptides identified from PilE (NMB_0018) after IP with α-glycan antisera with p<0.05 (ions score >23).
(PDF)

Table S5 Peptides identified from MetQ (NMB_1946) after IP with α-glycan antisera with p<0.05 (ions score >23).
(PDF)

Table S6 AniA-FLAG glycosylation site sequence variants.
(PDF)

Text S1. (PDF)
