# Supplementary figures and images for: Identification of Bacterial Protein O-Oligosaccharyltransferases and Their Glycoprotein Substrates
Source: PLoS One. 2013 May 3;8(5):e62768. doi: 10.1371/journal.pone.0062768 (PMC3643930; doi:10.1371/journal.pone.0062768)

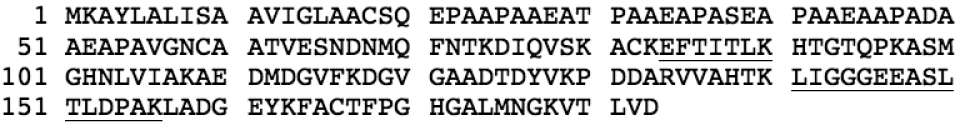

Supplement: Figure S1 — Peptide mapping coverage of Azurin (NMB_1533) after IP with α-glycan antisera. Peptides identified with p<0.05 (ions score >23) are underlined. (TIF) [file pone.0062768.s001.tif]

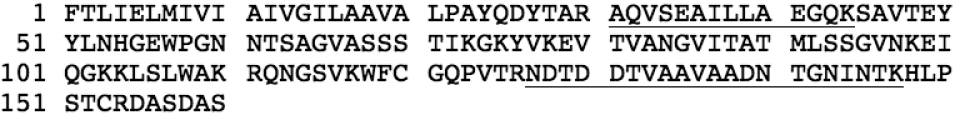

Supplement: Figure S2 — Peptide mapping coverage of PilE (NMB_0018) after IP with α-glycan antisera. Peptides identified with p<0.05 (ions score >23) are underlined. (TIF) [file pone.0062768.s002.tif]

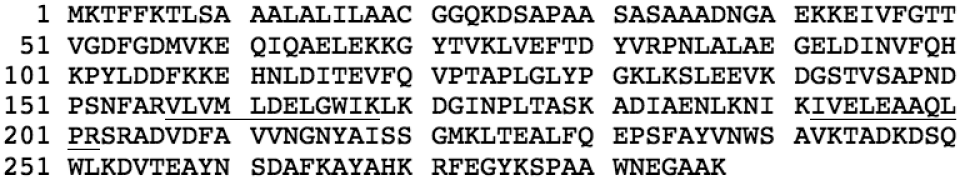

Supplement: Figure S3 — Peptide mapping coverage of MetQ (NMB_1946) after IP with α-glycan antisera. Peptides identified with p<0.05 (ions score >23) are underlined. (TIF) [file pone.0062768.s003.tif]

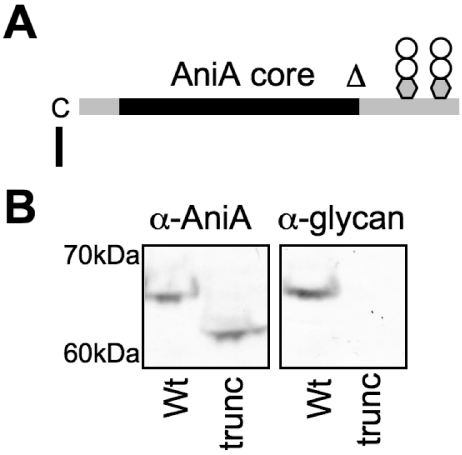

Supplement: Figure S4 — N. meningitidis AniA glycosylation (A) Cartoon of domains of N. meningitidis AniA protein showing: lipid-anchored N-terminal cysteine; N-terminal flexible region; AniA core fold; glycosylated C-terminal flexible region; Δ, truncated variant at Met354. (B) Western blots of FLAG-tagged purified AniA: wild type and C-terminally truncated (at Met 354, Δ in (A)), detected with either anti-FLAG or anti-glycan antisera. (TIF) [file pone.0062768.s004.tif]

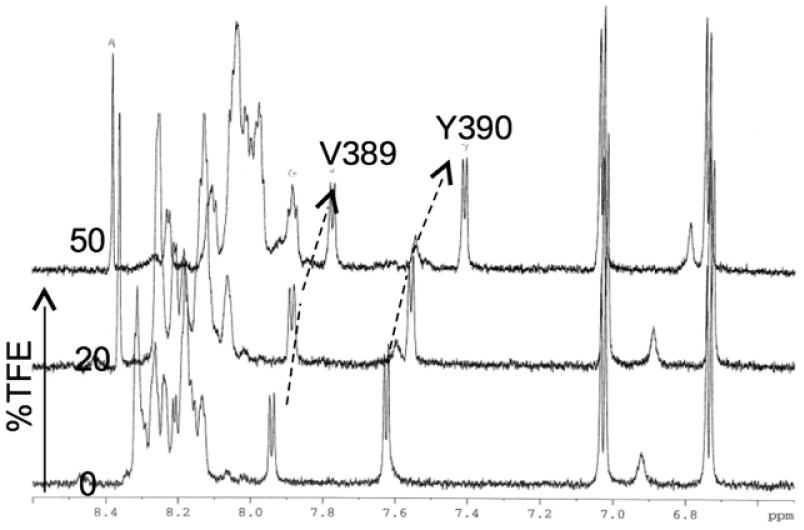

Supplement: Figure S5 — 750 MHz NMR spectra of the AniA glycosylation peptide in increasing concentrations of TFE-d6 (in 20 mM KPi, pH 6.5). The amide and aromatic region is shown (6.6–8.6 ppm). Increasing TFE-d6 results in upfield and downfield shifts of amide resonances. The shift of V389 and Y390 are shown dashed. (TIF) [file pone.0062768.s005.tif]
