# Supplementary material for: Identification of Bacterial Protein O-Oligosaccharyltransferases and Their Glycoprotein Substrates
Source: PLoS One. 2013 May 3;8(5):e62768. doi: 10.1371/journal.pone.0062768 (PMC3643930; doi:10.1371/journal.pone.0062768)
Supplement: Table S1 — Peptide sequences used for conjugate to Keyhole Limpet Hemocyanin to raise protein-specific antisera. (PDF) [file pone.0062768.s006.pdf]

Table S1.

Target protein	Peptide Sequence
ComP	AGALVAGTPSTAGSSCVGVQE
CcoP	HFLDKDKLHIMTAYV
MetQ	ENLKNIKIVELEAAQ
Sco	GGQNLPVIKQQYRVV
Mip	IGPNATLVFDVKLV
Laz	TDYVKPDDARVVAHT
