# Supplementary material for: Identification of Bacterial Protein O-Oligosaccharyltransferases and Their Glycoprotein Substrates
Source: PLoS One. 2013 May 3;8(5):e62768. doi: 10.1371/journal.pone.0062768 (PMC3643930; doi:10.1371/journal.pone.0062768)
Supplement: Table S2 — Proteins identified from N. meningitidis after IP with α-glycan antisera. (PDF) [file pone.0062768.s007.pdf]

Table S2.

Protein	Name	Ordered locus name	Uniprot Accession	Peptide coverage	MASCOT score
Azurin	Laz, azu	NMB_1533	F0AG13	12 %	428
Pilus structural subunit	PilE, pilin	NMB_0018	Q57135	21%	344
D-methionine binding protein	MetQ, Gna1946	NMB_1946	F0A283	8 %	269
