# Supplementary material for: Identification of Bacterial Protein O-Oligosaccharyltransferases and Their Glycoprotein Substrates
Source: PLoS One. 2013 May 3;8(5):e62768. doi: 10.1371/journal.pone.0062768 (PMC3643930; doi:10.1371/journal.pone.0062768)
Supplement: Table S3 — Peptides identified from Azurin (NMB_1533) after IP with α-glycan antisera with p<0.05 (ions score >23). (PDF) [file pone.0062768.s008.pdf]

Table S3.

Start-end	Observed (m/z)	Observed (Da)	ΔMass (Da)	Sequence	Ions scores
84-90	426.24^{2+}	850.47	-0.01	K.EFTITLK.H	43
141-156	524.28^{3+}	1569.83	0.00	K.LIGGGEEASLTLDPAK.L	82
141-156	785.93^{2+}	1569.85	0.02	K.LIGGGEEASLTLDPAK.L	139
