# Supplementary material for: Identification of Bacterial Protein O-Oligosaccharyltransferases and Their Glycoprotein Substrates
Source: PLoS One. 2013 May 3;8(5):e62768. doi: 10.1371/journal.pone.0062768 (PMC3643930; doi:10.1371/journal.pone.0062768)
Supplement: Table S4 — Peptides identified from PilE (NMB_0018) after IP with α-glycan antisera with p<0.05 (ions score >23). (PDF) [file pone.0062768.s009.pdf]

Table S4.

Start-end	Observed (m/z)	Observed (Da)	ΔMass (Da)	Sequence	Ions scores
31-44	486.27^{3+}	1455.79	-0.00	R.AQVSEAILLAEGQK.S	56
31-44	728.91^{2+}	1455.81	0.02	R.AQVSEAILLAEGQK.S	114
127-147	707.34^{3+}	2118.99	0.03	R.NDTDDTVAAVAADNTGNINTK.H	108
