# Supplementary material for: Identification of Bacterial Protein O-Oligosaccharyltransferases and Their Glycoprotein Substrates
Source: PLoS One. 2013 May 3;8(5):e62768. doi: 10.1371/journal.pone.0062768 (PMC3643930; doi:10.1371/journal.pone.0062768)
Supplement: Table S5 — Peptides identified from MetQ (NMB_1946) after IP with α-glycan antisera with p<0.05 (ions score >23). (PDF) [file pone.0062768.s010.pdf]

Table S5.

Start-end	Observed (m/z)	Observed (Da)	ΔMass (Da)	Sequence	Ions scores
157-168	708.41^{2+}	1414.79	0.02	R.VLVMLDELGWIK.L	95
192-202	61986^{2+}	1237.70	0.01	K.IVELEAAQLPR.S	86
