# Supplementary material for: Identification of Bacterial Protein O-Oligosaccharyltransferases and Their Glycoprotein Substrates
Source: PLoS One. 2013 May 3;8(5):e62768. doi: 10.1371/journal.pone.0062768 (PMC3643930; doi:10.1371/journal.pone.0062768)
Supplement: Table S6 — AniA-FLAG glycosylation site sequence variants. (PDF) [file pone.0062768.s011.pdf]

Table S6.

Variant	Leu358-Lys387 Peptide Sequence
WT	LSDTAYAGNGAAPAASAPAASAPAASASEK
S373A	LSDTAYAGNGAAPAA**A**APAASAPAASASEK
S378A	LSDTAYAGNGAAPAASAPAA**A**APAASASEK
S373A, S378A	LSDTAYAGNGAAPAA**A**APAA**A**APAASASEK
S373A, S378A, S385P	LSDTAYAGNGAAPAA**A**APAA**A**APAASA**P**EK

Underlined S are glycosylated, bold **A** and **P** are changed in variants
