# Supplementary material for: Identification of Bacterial Protein O-Oligosaccharyltransferases and Their Glycoprotein Substrates
Source: PLoS One. 2013 May 3;8(5):e62768. doi: 10.1371/journal.pone.0062768 (PMC3643930; doi:10.1371/journal.pone.0062768)
Supplement: Text S1. — (PDF) [file pone.0062768.s012.pdf]

Supporting Information Text

The similarities between the *O*-glycosylation of pili and proteins in *N. meningitidis* and the *wzy*-dependent biosynthesis of O-antigen in *E. coli* have previously been reported [1,2]. A key point at which these pathways diverge is the ligase or *O*-OTase. In protein glycosylation, the *O*-OTase adds a glycan to the target protein, whereas in the O-antigen system the ligase adds an oligosaccharide to the core-LPS. Indeed these pathways are so similar and conserved between species that the expression of *P. aeruginosa* PilO and *N. meningitidis* PglL *O*-OTases and their target proteins in an *E. coli* strain with an intact O-antigen biosynthesis system results in the addition of a single O-antigen unit to the target protein [1]. In some systems the biosynthesis pathways serve both functions providing oligosaccharides for both O-antigen and protein glycosylation [3]. Thus, the identification of homologues of O-antigen or *O*-OTase biosynthesis genes in a genome does not enable the functional categorization of these genes. The glycosylation systems thus far described add single units of an O-antigen even when working O-antigen polymerases and chain length determinants are present, whereas WaaL homologues generally add the polymerised O-antigen. The presence of O-antigen polymerase and chain length determinant in a genome indicates that the species may have an O-antigen but the absence of these genes does not indicate that O-antigen/O-OTase genes are not LPS genes since some species do not have O-antigens but have a wzy-dependent system which adds a single oligosaccharide to the core-LPS [4]. Therefore, the key gene differentiating these two systems is the ligase/*O*-OTase. These genes have low levels of amino acid sequence similarity and are not differentiated by available HMMs.

Two protein O-glycosylation O-antigen-like *O*-OTases were known at the beginning of this study; PglL (NMA0800) [2] and PilO (*P. aeruginosa*). The PFAM database places PglL in the PF04932 family. This protein family is wrongly annotated in the PFAM database as being an O-antigen polymerase family (a mistake in annotation that has been perpetuated from the PFAM database into the automatic annotation of many bacterial genomes). Analysis using the *wzy* gene (PID:1197647) which is described as the seed of this PFAM [5] (http://pfam.sanger.ac.uk/family?acc=PF04932) reveals that this HMM does not match chain length determinates as described. We suggest that the PFAM domain which describes the family is contained in O-antigen ligases and O-glycosylation *O*-OTases. The PglL motif HMMs are found in a minority of the members of the PF04932 family (62/554 of the proteins identified http://PFAM.sanger.ox.ac.uk).

Supporting Information Text References

1. Faridmoayer A, Fentabil MA, Mills DC, Klassen JS, Feldman MF (2007) Functional characterization of bacterial oligosaccharyltransferases involved in O-linked protein glycosylation. J Bacteriol 189: 8088-8098.
2. Power PM, Seib KL, Jennings MP (2006) Pilin glycosylation in Neisseria meningitidis occurs by a similar pathway to wzy-dependent O-antigen biosynthesis in Escherichia coli. Biochem Biophys Res Commun 347: 904-908.
3. Tabei SM, Hitchen PG, Day-Williams MJ, Merino S, Vart R, et al. (2009) An Aeromonas caviae genomic island is required for both O-antigen lipopolysaccharide biosynthesis and flagellin glycosylation. J Bacteriol 191: 2851-2863.
4. Hood DW, Randle G, Cox AD, Makepeace K, Li J, et al. (2004) Biosynthesis of cryptic lipopolysaccharide glycoforms in Haemophilus influenzae involves a mechanism similar to that required for O-antigen synthesis. J Bacteriol 186: 7429-7439.
5. Bengoechea JA, Pinta E, Salminen T, Oertelt C, Holst O, et al. (2002) Functional characterization of Gne (UDP-N-acetylglucosamine-4-epimerase), Wzz (chain length determinant), and Wzy (O-antigen polymerase) of Yersinia enterocolitica serotype O:8. J Bacteriol 184: 4277-4287.
6. Faridmoayer A, Fentabil MA, Florencia Haurat M, Yi W, Woodward R, et al. (2008) Extreme substrate promiscuity of the Neisseria oligosaccharyltransferase involved in protein O-glycosylation. J Biol Chem 283: 34596-34604.
